# Toward a better understanding of the mechanisms of symbiosis: a comprehensive proteome map of a nascent insect symbiont

**DOI:** 10.7717/peerj.3291

**Published:** 2017-05-09

**Authors:** François Renoz, Antoine Champagne, Hervé Degand, Anne-Marie Faber, Pierre Morsomme, Vincent Foray, Thierry Hance

**Affiliations:** 1 Biodiversity Reasearch Center, Université catholique de Louvain, Louvain-la-Neuve, Belgium; 2 Institute of Life Sciences, Université catholique de Louvain, Louvain-la-Neuve, Belgium; 3 Centre de Recherche de Biochimie Macromoléculaire, Centre National de la Recherche Scientifique, Montpellier, France

**Keywords:** Proteome, *Serratia symbiotica*, Symbiosis, Iron metabolism, Symbiotic factors

## Abstract

Symbiotic bacteria are common in insects and can affect various aspects of their hosts’ biology. Although the effects of insect symbionts have been clarified for various insect symbiosis models, due to the difficulty of cultivating them *in vitro*, there is still limited knowledge available on the molecular features that drive symbiosis. *Serratia symbiotica* is one of the most common symbionts found in aphids. The recent findings of free-living strains that are considered as nascent partners of aphids provide the opportunity to examine the molecular mechanisms that a symbiont can deploy at the early stages of the symbiosis (i.e., symbiotic factors). In this work, a proteomic approach was used to establish a comprehensive proteome map of the free-living *S. symbiotica* strain CWBI-2.3^T^. Most of the 720 proteins identified are related to housekeeping or primary metabolism. Of these, 76 were identified as candidate proteins possibly promoting host colonization. Our results provide strong evidence that *S. symbiotica* CWBI-2.3^T^ is well-armed for invading insect host tissues, and suggest that certain molecular features usually harbored by pathogenic bacteria are no longer present. This comprehensive proteome map provides a series of candidate genes for further studies to understand the molecular cross-talk between insects and symbiotic bacteria.

## Introduction

Insecta is the most diverse class of animals described so far, and many of them are intimately associated with symbiotic bacteria (Buchner, 1965). Some symbionts are obligate partners for their host and fulfill an essential nutritional function (Baumann, 2005; Vigneron et al., 2014), whereas others are considered as facultative symbionts by being only beneficial in the context of various ecological interactions (Oliver et al., 2010). Regarding these relationships between insects and bacteria, particular attention has been paid in the recent decades to the symbionts harbored by members of the *Aphididae* family (Baumann, 2005; Oliver et al., 2010). In order to get essential amino acids, these sap-feeding insects rely on their nutritional obligate symbiont *Buchnera aphidicola* which is confined to a specialized organ called the bacteriome (Moran, McCutcheon & Nakabachi, 2008). In addition to *B. aphidicola*, aphids can also harbor a wide range of facultative symbionts that are not essential but can bring benefits to their hosts according to environmental conditions, such as protection against parasites and heat-stress, and adaptation to host plants (Oliver et al., 2010).

Ultimately derived from free-living bacterial ancestors, symbiotic bacteria share similar mechanisms with opportunistic pathogens to facilitate successful colonization of a particular niche (Hentschel, Steinert & Hacker, 2000). These mechanistic similarities for host colonization are particularly prominent in facultative symbionts (Dale & Moran, 2006; Silver et al., 2007; Degnan et al., 2009; Watson et al., 2010) considering their short-time association with insects in comparison to obligate symbionts that lead to the loss of many genes during the acquisition of the endosymbiotic lifestyle (Moran, McCutcheon & Nakabachi, 2008). And unlike obligate symbionts that display a strict intracellular lifestyle, facultative ones exhibit a more flexible tissue tropism, ranging from intracellular localization within specialized host cells (e.g., bacteriocytes) to extracellular lifestyle in host gut or other tissues (Salem et al., 2015). This more flexible tissue tropism is expected to facilitate horizontal transfers of facultative symbionts beside their maternal transmission (Sandström et al., 2001). Host acquisition of facultative partners from various environmental sources indicates that they have retained the ability to infect alternative hosts and are more prone to survive outside their primary host in comparison to obligate symbionts (Renoz et al., 2015; Salem et al., 2015).

While the beneficial effects of facultative symbionts have been clarified in a number of insect symbiosis models (Oliver et al., 2010; Feldhaar, 2011), the molecular features underlying initiation and establishment of such associations remain elusive. With regard to other models of symbiosis between microbes and animals or plants (Bright & Bulgheresi, 2010; Oldroyd, 2013; Kremer et al., 2013), there is no doubt that the establishment and persistence of these facultative partnerships in insects are determined by complex molecular cross-talks between both partners. This so-called “symbiotic partnership” involves colonization, invasion, and host modulation factors displayed by the bacterial partners, such as quorum sensing systems, iron chelators, secretion and transport mechanisms that can interfere directly with host cellular function and coordinate behavior of symbiotic bacteria for priming host invasion (Hentschel, Steinert & Hacker, 2000). Considering the evolutionary perspective, symbiotic bacteria may derive from opportunistic pathogens through several steps that include the loss of virulence factors that may harm the host while keeping functional the genes allowing host colonization in the context of mutual benefits (Ochman & Moran, 2001).

The limited understanding of the bacterial mechanisms that prime initiation of the symbiosis is mainly due to the difficulty of isolating them from their insect hosts and cultivating them *in vitro* because of their host dependence (Kikuchi, 2009). Recently, culturing attempts have been successful for several strains of *Serratia symbiotica* (Sabri et al., 2011; Foray et al., 2014), one of the most common facultative symbionts found in aphids (Oliver et al., 2010). This symbiont species exhibits a wide diversity of strains that vary in their degree of reliance on hosts, ranging from co-obligate and strictly intracellular strains (Manzano-Marín & Latorre, 2016) to free-living extracellular strains residing in the insect gut (F Renoz, I Pons, G Bataille, C Noël, V Foray, V Pierson & T Hance, 2015, unpublished data). Genomic features of the strain CWBI-2.3^T^, one of these so-called “free-living” *S. symbiotica*, suggest that these strains are involved in the nascent phases of the symbiosis (Manzano-Marín & Latorre, 2016), and could be acquired directly from environmental sources to form emerging symbiotic associations with aphid partners (Renoz et al., 2015).

*S. symbiotica* strain CWBI-2.3^T^ offers a unique opportunity to analyze what remains of the molecular machinery that is involved at the beginning of an evolutionary transition from an independent lifestyle in the environment to a more intimate lifestyle. In the present study, we used a gel-free proteomic approach applied to symbiont cell cultures in order to decipher the molecular features that a free-living *S. symbiotica* holds and can express to initiate host invasion, without being a pathogen (Renoz et al., 2015). Taking advantage of the *S. symbiotica* CWBI-2.3^T^ genome sequence (Foray et al., 2014), we report here the characterization of the membrane and cytosolic proteomes of this symbiont. 720 different proteins have been identified, and classified by their putative function. Of these, 76 were identified as putative symbiotic factors. Ours results provide strong evidence the *S. symbiotica* CWBI-2.3^T^ is well-armed to invade the insect tissues, and suggest that certain molecular features usually found in pathogenic bacteria are no longer present. This comprehensive proteome map of an insect facultative symbiont provides a first step and a solid basis to implement genetic modification experiments to tackle the mechanisms used by symbiotic bacteria to settle in a novel insect host.

## Materials and Methods

### Bacterial strain and growth conditions

A free-living *S. symbiotica* strain, CWBI-2.3^T^, isolated from a natural *Aphis fabae* collected in Belgium in 2009 was routinely maintained on 863 agar at 20 °C, containing 1% glucose, 1% yeast extract, 1% casein peptone, and 1.7% agar. A draft version of the *S. symbiotica* CWBI-2.3^T^ genome is available under the GenBank accession number CCES01000000. This draft contains 3,664 predicted protein-coding sequences (Foray et al., 2014).

To prepare protein extracts, triplicate cultures were grown in 400 mL of 863 medium under vigorous agitation at 20 °C (Sabri et al., 2011) and were collected during the exponential growth phase when reaching an optical density (OD) of 0.4–0.6 at 600 nm. Cells were harvested by centrifugation at 3,200 × g for 10 min at 20 °C. The resulting pellets were washed with cooled sterile PBS (pH 7.4) and stored at −80 °C until protein extraction.

### Protein extraction

Bacterial cells were resuspended in 500 µL of homogenization buffer (100 mM TEAB, 1mM PMSF, and 2 mg mL^−1^ each of leupeptin, aprotinin, antipain, pepstatin, and chymostatin). Samples were sonicated three times at high intensity for 5 min at 4 °C using a bath sonicator (Bioruptor, Diagenode). Cellular wastes were removed by a 2,000 rpm centrifugation of 5 min at 4 °C. The supernatant was then centrifuged at 4 °C for 30 min at 40,000 rpm to separate the microsome fraction from the soluble fraction. The protein concentration was determined by Bradford assay (Bradford, 1976) using a commercial dye reagent (Bio-Rad, Hercules, CA, USA) and using IgG gamma globulin as a standard. Both the soluble and the microsome fractions were subjected to chloroform/methanol precipitation, as previously described (Wessel & Flügge, 1984).

### Reduction/alkylation/digestion

Proteins were suspended in 100 µL of 50 mM NH_4_HCO_3_ containing 0.1% RapiGest (Waters) for soluble fraction and 0.5% RapiGest for crude membranes by vortexing at room temperature for 30 min and by sonicating at high intensity for 5 min at 4 °C using a bath sonicator (Bioruptor; Diagenode, Seraing, Belgium). Disulfide bonds were reduced by incubation for 1 h at 60 °C with 25 mM tris(2-carboxyethyl)phosphine. Then, cysteine residues were blocked in 200 mM methyl-methanethiosulfonate (MMTS) for 15 min at room temperature in the dark. Solubilized crude membrane proteins were first diluted five times with 50 mM TEAB to reach a concentration of 0.1% (v/v) in RapiGest. Protein digestions was performed overnight at 37 °C using sequencing-grade-modified trypsin (Promega, Madison, WI, USA) at a protease/protein ratio of 1/20 and RapiGest subsequently lysed by incubating the protein sample in 1% trifluoroacetic acid (TFA) for 1 h at 37 °C. After centrifugation of the sample at 54,000 rpm for 45 min at 4 °C, the supernatant was vacuum dried (SpeedVac SC 200, Savant).

### 1-D LC separation

Samples (20 µg) were resuspended in loading buffer (2% ACN, 0.1% TFA) and subjected to reverse-phase chromatography on a C18 PepMap 100 column (Acclaim^®^ pepMap 100-75 µM i.d.  × 5 mm-C18-3 µm-100 Å; LC Packing, Sunnyvale, CA, USA) for 180 min at a flow rate of 300 nL min^−1^ using a linear gradient from 8% (v/v) ACN in water/0.1% (v/v) TFA to 76% (v/v) ACN in water/0.1% (v/v) TFA. The eluted peptides were spotted onto a MALDI plate together with the ionization matrix (4 mg mL^−1^ of CHCA, 70% ACN, 0.1% TFA) (Probot; LC Packings, Sunnyvale, CA, USA).

### MALDI-MS/MS and database search analysis

Mass spectrometry analyses were performed on an AB 4800 MALDI TOF/TOF analyzer using a 200 Hz solid-state laser operating at 355 nm (Szopinska et al., 2011). MS spectra were obtained using a laser intensity of 3,200 and 2,000 laser shots per spot (100 shots/sub-spectrum) in the *m/z* range of 800–4,000, whereas MS/MS spectra were obtained by automatic selection of the 15 most intense precursor ions per spot using a laser intensity of 4,000 and 2,000 laser shots per precursor (100 shots/sub-spectrum). Collision-induced dissociation was performed with an energy of 1 kV with air as the collision gas at a pressure of 1.10^6^ Torr.

Data were collected using the ABSciex 4000 Series Explorer™ software. MS data from the soluble and the microsome fractions were pooled and analyzed as a single data set using ProteinPilot software v.4.0 with the Paragon™ search algorithm (Shilov et al., 2007) (AB SCIEX). The MS data were searched against the *S. symbiotica* CWBI-2.3^T^ database (containing 3,397 protein sequences downloaded on Augustus 08th 2015, GenBank accession number: CCES00000000). The searched options in ProteinPilot were “thorough search”, “MMTS” as the cysteine modification, “trypsin” as the digestion enzyme. All reported proteins were identified with 95% or greater confidence, as determined by ProteinPilot unused scores (>1.3). This corresponds to a stringent threshold of false discovery rate, lower than 1%. The “unused” score is a measurement of the protein identification confidence taking into account peptides from spectra that have not already been used by higher scoring proteins.

All identified proteins were analyzed by PSORTb version 3.0.2 (http://www.psort.org/psortb/; Gardy et al., 2005) for *in silico* analysis of their localization and were assigned to functional categories based on clusters of orthologous group of proteins (COG; http://www.ncbi.nlm.nih.gov/COG/).

### Phenotypic assays

#### Detection of siderophores

Siderophore systems enable invasive bacteria to scavenge iron under limiting conditions in symbiosis (Braun & Hantke, 2011; Han et al., 2013). Here, Chromazurol (CAS, Schwyn & Neilands, 1987) agar was employed to test the production of siderophores by *S. symbiotica* CWBI-2.3^T^. On CAS agar plates a color change from blue to orange indicates siderophore producing bacteria due to Fe^3+^ removal from the dye. *Bacillus subtilis*, a siderophore-producing bacterial species, was used as positive control while *Bacillus pumilus* served as negative control.

#### Swimming motility testing of S. symbiotica CWBI-2.3^**T**^

Motility is a bacterial function that can be required for host colonization (Lee et al., 2015). Swimming motility testing was conducted according to a previously described procedure with a slight modification (Picot et al., 2004). Briefly, Semisolid Motility Test medium was used to detect motility. The agar concentration is sufficient to form a soft gel without hindering motility. Precultures of bacterial isolates were separately prepared in 863 medium during the exponential growth phase (OD_600_ of 0.4–0.6), and layered on plates with 863 medium containing 1.7% agar. The plates were incubated at 20 °C until colonies developed. Swimming motility was evaluated using plates prepared with 863 medium supplemented with 0.2% agar. A single colony was inoculated by puncture in the middle of the plates. The motility was estimated by measuring the diameter of the halo (in centimeters) four days after inoculation. Due to it swimming motility capacity, *S. marcescens* Db11 was used as positive control while *Staphylococcus xylosus* was used as negative control. For every result, the value of mobility was determined on three independent replicates. Results were expressed as mean ± standard error of the mean (SEM).

## Results and Discussion

### Proteome map

The reference proteome map was built through the analysis of the soluble and the microsome fractions from three independent *S. symbiotica* CWBI-2.3^T^ cultures using a gel-free approach. Due to their low abundance and poor solubility, membrane proteins are generally poorly represented on two-dimensional gels (Geert Baggerman, 2006). We therefore decided to rely on a gel-free approach. Soluble and membrane proteins were separated by ultracentrifugation and the microsome fraction was stripped at alkaline pH to remove soluble proteins (Wu et al., 2003). Overall, spectral data were pooled and analyzed as a single data set. This led to the identification of 720 different proteins (Table S1), corresponding to 19.7% of all theoretically expressed proteins of the recently sequenced strain *S. symbiotica* CWBI-2.3^T^ (Foray et al., 2014). This percentage of identified proteins reflects the proteins effectively expressed in our experimental conditions. Nevertheless, despite the experimental approach we used to identify as many hydrophobic membrane proteins as possible, some proteins cannot be efficiently detected because of their low-abundance and the limitations of the analytical methods (Garbis, Lubec & Fountoulakis, 2005).

The *in silico* prediction of the cellular localization of these identified proteins suggests that our proteome map consisted of 64% cytoplasmic proteins, 3.5% outer membrane proteins (OMPs), 2.4% periplasmic proteins, 17% inner membrane proteins (IMPs) and less than 1% extracellular proteins. It was not possible to predict the exact cellular localization for 12.4% of the identified proteins (either multiple localization sites or unknown localization).

**Figure 1:**
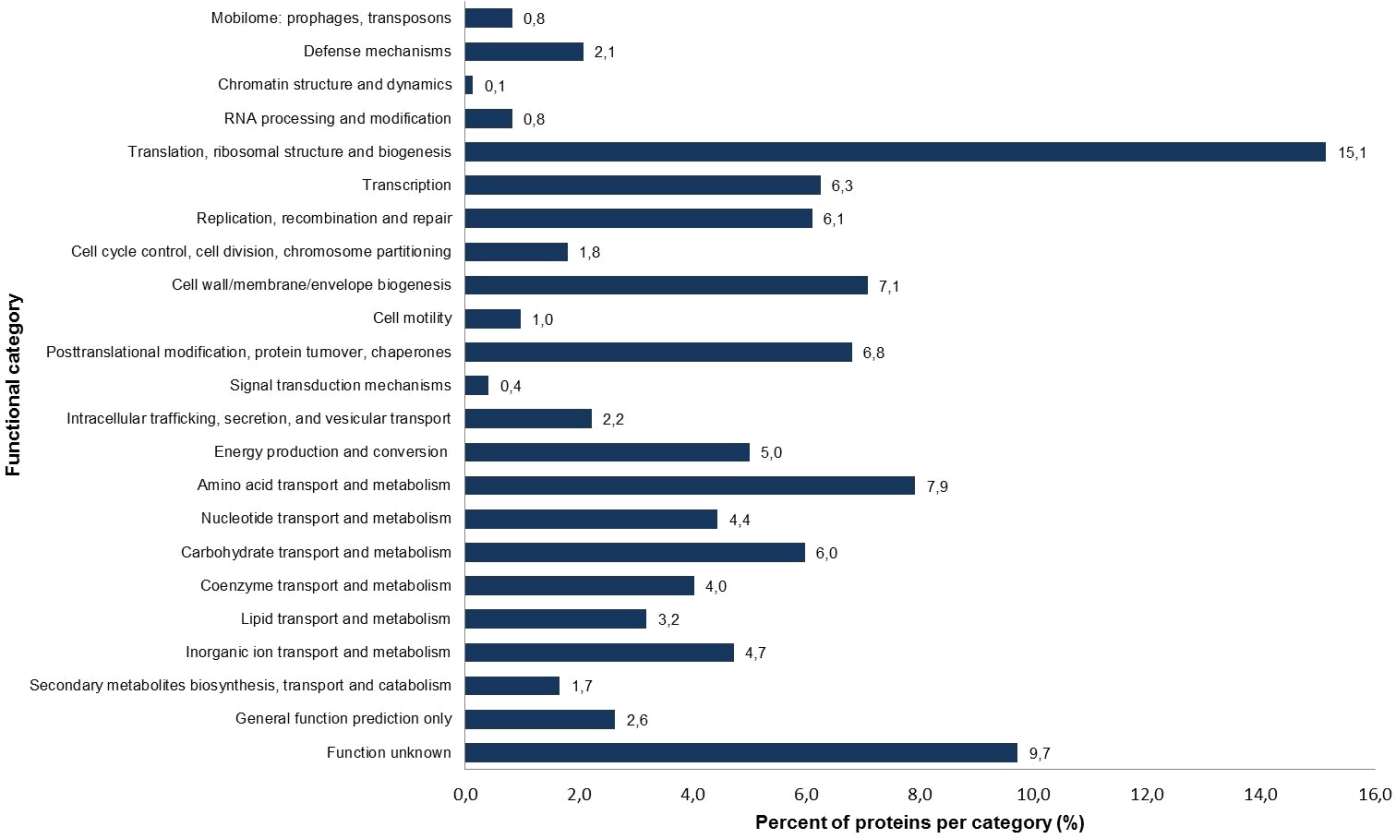
Percentage of identified proteins according to different functional categories based on the NCBI COG functional annotation.

**Table 1:** Identification of putative symbiotic factors in *S. symbiotica* that might be required for establishing an association with its insect host.

Protein designation	Accession number	Predicted localization
Motility and chemotaxis		
Flagellar L-ring protein	CDS58522.1	Outer membrane
Flagellar P-ring protein	CDS58521.1	Periplasmic
Basal-body rod modification protein FlgD	CDS58526.1	Extracellular
Flagellar basal-body rod protein FlgG	CDS58523.1	Extracellular
Flagellar biosynthesis protein FlhA	CDS58536.1	Inner membrane
Flagellar brake protein YcgR	CDS58313.1	Cytoplasmic
Flagellar transcriptional regulator FlhC	CDS58540.1	Cytoplasmic
Flagellar transcriptional regulator FlhD	CDS58541.1	Cytoplasmic
Probable HTH-type transcriptional regulator LrhA	CDS57959.1	Cytoplasmic
Type IV pilus biogenesis and competence protein PilQ	CDS55476.1	Outer membrane
Adhesion, invasion, biofilm formation		
Transcriptional regulatory protein PhoP	CDS58188.1	Cytoplasmic
Sensor protein PhoQ	CDS58187.1	Inner membrane
Phosphate-specific transport system accessory protein PhoU	CDS56284.1	Cytoplasmic
Transcriptional regulatory protein CpxR	CDS56277.1	Cytoplasmic
Transcriptional regulatory protein OmpR	CDS56155.1	Cytoplasmic
Osmolarity sensor protein EnvZ	CDS56154.1	Inner membrane
Acyl-homoserine-lactone synthase SwrI	CDS56499.1	Cytoplasmic
cAMP-activated global transcriptional regulator CRP	CDS56109.1	Cytoplasmic
DNA-binding protein H-NS	CDS58425.1	Cytoplasmic
RNA polymerase sigma factor RpoS	CDS55654.1	Cytoplasmic
Outer membrane protein A	CDS57861.1	Outer membrane
FKBP-type peptidyl-prolyl cis-trans isomerase FkpA	CDS56095.1	Periplasmic
Iron acquisition		
Biopolymer transport protein ExbB	CDS55934.1	Inner membrane
Biopolymer transport protein ExbD	CDS55935.1	Inner membrane
Ferric uptake regulation protein	CDS57198.1	Cytoplasmic
Periplasmic chelated iron-binding protein YfeA	CDS58219.1	Periplasmic
Chelated iron transport system membrane protein YfeB	CDS58218.1	Inner membrane
Bacterial non-heme ferritin	CDS58146.1	Cytoplasmic
DNA protection during starvation protein	CDS57357.1	Cytoplasmic
Putative peroxiredoxin bcp	CDS57424.1	Unknown
Succinate dehydrogenase flavoprotein subunit	CDS57210.1	Inner membrane
Succinate dehydrogenase iron-sulfur subunit	CDS57211.1	Inner membrane
Fe(3+) dicitrate transport protein FecA	CDS55609.1	Outer membrane
Protection against reactive oxygen radicals		
Superoxide dismutase [Mn]	CDS56813.1	Probable multiple localization sites
Catalase	CDS57987.1	Probable multiple localization sites
Thiol peroxidase	CDS58350.1	Periplasmic
Peroxiredoxin Bcp	CDS57424.1	Unknown
Delta-aminolevulinic acid dehydratase HemB	CDS56723.1	Cytoplasmic
Porphobilinogen deaminase HemC	CDS56648.1	Cytoplasmic
Uroporphyrinogen-III C-methyltransferase HemX	CDS56650.1	Inner membrane
Glutamate-1-semialdehyde 2,1-aminomutase HemL	CDS55589.1	Cytoplasmic
Protein HemY	CDS56651.1	Inner membrane
Putative thioredoxin domain-containing protein	CDS58953.1	Cytoplasmic
Glutaredoxin YdhD	CDS58265.1	Unknown
Glutathione reductase	CDS56214.1	Cytoplasmic
Electron transport complex subunit C	CDS58281.1	Cytoplasmic
Thioredoxin reductase	CDS57678.1	Cytoplasmic
Hydrogen peroxide-inducible genes activator	CDS56327.1	Cytoplasmic
Thioredoxin	CDS56739.1	Cytoplasmic
Chaperone protein ClpB	CDS59016.1	Cytoplasmic
Chaperone protein DnaK	CDS55519.1	Cytoplasmic
Protein GrpE	CDS58735.1	Cytoplasmic
Chaperone protein HtpG	CDS58965.1	Cytoplasmic
Fe/S biogenesis protein NfuA	CDS56165.1	Cytoplasmic
Secretion and transport mechanisms		
Protein translocase subunit SecA	CDS55578.1	Cytoplasmic
Protein-export protein SecB	CDS56816.1	Cytoplasmic
Protein translocase subunit SecD	CDS58907.1	Inner membrane
Protein translocase subunit SecF	CDS58906.1	Inner membrane
Protein-export membrane protein SecG	CDS56777.1	Inner membrane
Protein translocase subunit SecY	CDS56065.1	Inner membrane
Sec-independent protein translocase protein TatA	CDS56720.1	Inner membrane
SecYEG protein translocase auxillary subunit	CDS58908.1	Inner membrane
Membrane protein insertase YidC	CDS56458.1	Inner membrane
Type II secretion system protein G	CDS55483.1	Inner membrane
Similar to Syringopeptin synthetase C (fragment)	CDS58510.1	Probable multiple localization sites
Multidrug efflux pump subunit AcrA	CDS58973.1	Inner membrane
Outer membrane protein TolC	CDS55974.1	Outer membrane
Hydrolytic enzymes		
Chaperone protein ClpB	CDS59016.1	Cytoplasmic
ATP-dependent Clp protease ATP-binding subunit ClpX	CDS58879.1	Cytoplasmic
ATP-dependent Clp protease proteolytic subunit	CDS58880.1	Cytoplasmic
Lon protease	CDS58878.1	Cytoplasmic
Periplasmic serine endoprotease DegP	CDS55594.1	Periplasmic
Periplasmic pH-dependent serine endoprotease DegQ	CDS55928.1	Periplasmic
Zinc metallopeptidase	CDS58788.1	Inner membrane
ATP-dependent zinc metalloprotease FtsH	CDS56781.1	Inner membrane
Chitodextrinase (modular protein)	CDS55463.1	Unknown

The *S. symbiotica* CWBI-2.3^T^ annotated proteins were classified into 23 clusters of orthologous group (COG) categories (Fig. 1). Most of the proteins belong to the following categories: translation (15.1%), amino acid transport and metabolism (7.9%), cell wall/membrane/envelope biogenesis (7.1%), replication/recombination and repair (6.1%) and carbohydrate transport and metabolism (6.0%). The putative symbiotic factors that have been identified in this study (summarized in Table 1) were classified according to their assumed function. Putative symbiotic traits are involved in: (i) motility and chemotaxis, (ii) adhesion, invasion and biofilm formation, (iii) iron uptake, (iv) protection against reactive oxygen radicals, (v) secretion and transport mechanisms, and (vi) hydrolytic enzymes.

**Figure 2:**
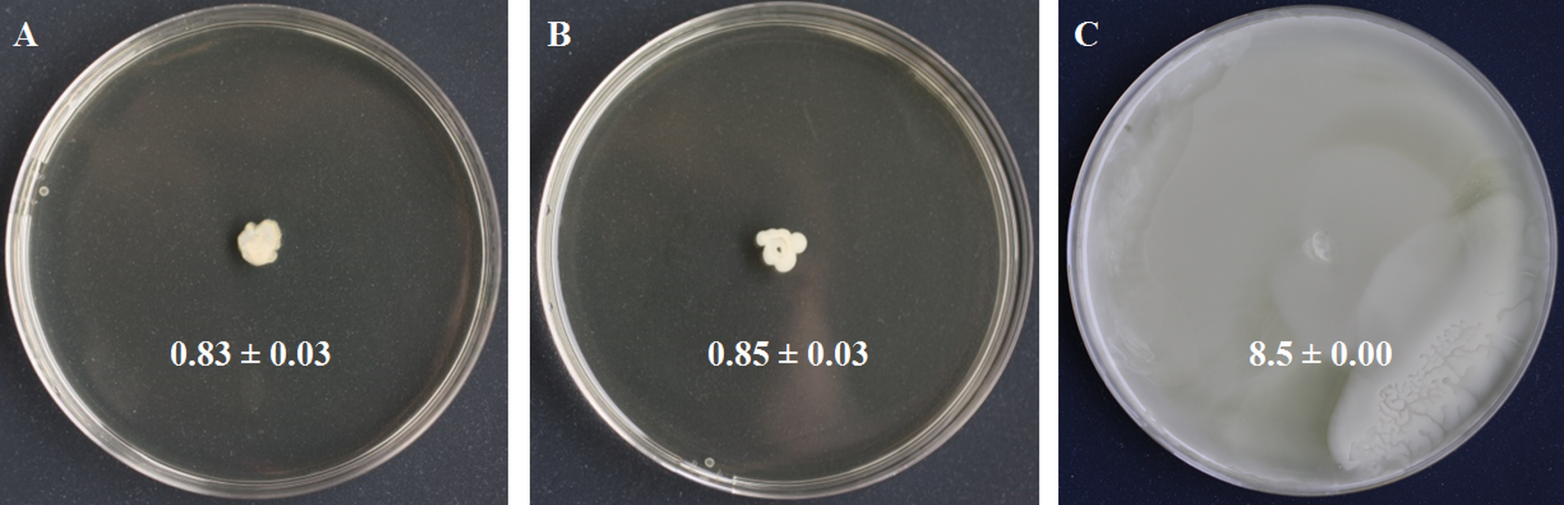
Swimming motility testing of *S. symbiotica* CWBI-2.3^T^ as described in Materials and methods. *S. symbiotica* is not endowed with swimming motility (A). *S. xylosus* was used as negative control (B) and *S. marcescens* Db11 was used as positive control (C).

### Motility and chemotaxis

Motility is a bacterial function often required for initiating host colonization, symbiont transmission and symbiosis establishment (Ruby, 2008; Lee et al., 2015). Motility is mostly driven by flagella made of filament, basal body and motor (Ridgway, Silverman & Simon, 1977). We identified four basal body proteins (FlgH, CDS58522.1; FlgI, CDS58521.1; FlgD, CDS58526.1; FlgG, CDS58523.1), one motor protein (FlhA, CDS58536.1), but no filamentous proteins. We also identified the protein YcgR (CDS58313.1), a member of the T3SS family that acts as a flagellar brake, regulating swimming and swarming by interaction with motor proteins (Paul et al., 2010). We detected FlhC (CDS58540.1) and FlhD (CDS58541.1) that form the major transcriptional regulator FlhDC of flagellum biosynthesis and whose expression depends on flagellar secretion apparatus component FlhA (CDS58536.1) that participates in the secretion of the hook and filament proteins (Bange et al., 2010). Finally, we identified LrhA (CDS57959.1), a transcriptional regulator of *flhDC* (Lehnen et al., 2002). In the light of these results, swimming motility was tested to know whether the flagellar apparatus of *S. symbiotica* CWBI-2.3^T^ was functional. The motility assay (Fig. 2) showed that *S. symbiotica* CWBI-2.3^T^ is not endowed with swimming motility in the conditions used in this study. Genes involved in cell motility are among the more highly reduced in the evolutionary transition from a free-living to an endosymbiotic lifestyle (Manzano-Marín et al., 2012). In obligate intracellular symbionts and many facultative ones, a significant number of genes responsible for flagellar assembly have been partially or totally lost during their evolutionary transition (Moya et al., 2008; Degnan et al., 2010), and only export proteins within the flagella assembly pathway have been kept (Toft & Fares, 2008). Consequently, these symbionts have become nonmotile. This seems to be the case for *S. symbiotica* CWBI-2.3^T^. Nevertheless, it cannot be excluded that *S. symbiotica* use other kinds of motility such as twitching since we identified PilQ (CDS55476.1), a secretin that is essential for the biogenesis of type IV pili and that can be involved in twitching motility, adhesion to host epithelial cells and in protein secretion (Lim et al., 2008).

Motility is generally pointed to as a bacterial function working together with chemotaxis, the capacity of motile bacteria to sense and respond to changes in the concentration of chemicals in the environment. By mediating bacterial migration into the host tissues, chemotaxis enables bacteria to reach and maintain their preferred niches for colonization (Caetano-Anollés, Crist-Estes & Bauer, 1988; Kremer et al., 2013). In our study, we did not identify chemotaxis (Che) proteins and chemoreceptor proteins (methyl-accepting chemotaxis protein [MCP]). The only chemotaxis-related protein that was identified is LrhA (CDS57959.1), which has been observed to be a key transcriptional regulator of flagella, motility and chemotaxis genes in *E. coli* (Lehnen et al., 2002). The *che* genes are absent from the genome sequence of *S. symbiotica* CWBI-2.3^T^ as well as from other *S. symbiotica* strains, while they are found in the other *Serratia* species and other insect facultative symbionts (Toh et al., 2006). The absence of *che* genes in *S. symbiotica* could also be a consequence of its progressive evolutionary transition from a free-living to an endosymbiotic life-style that involves the loss of flagellar proteins, fimbrial, pili and chemotaxis-related proteins dispensable in a stable environment (i.e., the insect tissues in which bacterial symbionts reside) (Manzano-Marín et al., 2012).

### Proteins involved in adhesion, invasion and biofilm formation

#### The two-component signaling systems

The two-component signaling systems (TCS) are among the most universal mechanism by which bacteria sense their environment and respond accordingly (Groisman, 2001). They have been pointed to as essential in the formation of biofilms that can protect invasive bacteria against antimicrobial agents, such as antibiotics and host immune effectors (Ramey et al., 2004; Kim et al., 2014). *S. symbiotica* CWBI-2.3^T^ encodes the PhoP/Q regulon (CDS58188.1 and CDS58187.1) that is associated with virulence in various bacterial pathogens (Groisman, 2001; García-Calderón, Casadesús & Ramos-Morales, 2007) and that regulates the activation of secretion system genes that are required for host cell invasion and intracellular survival (Dale & Moran, 2006). The mutualistic symbiont of tsetse flies, *Sodalis glossinidus* carrying a *phoP* mutant fails to infect insect hosts (Pontes et al., 2011), suggesting that the PhoP/Q system is necessary for host colonization and the establishment of a symbiotic association. *phoP/Q* is absent in the genome of several obligate and facultative symbionts (Hansen, Vorburger & Moran, 2012) which is probably a consequence of the transition lifestyle from an opportunistic to an obligate association that is accompanied by the loss of genes that no longer provide an adaptive benefit in a stable intracellular symbiotic relationship (Dale & Moran, 2006).

We also identified PhoU (CDS56284.1) which controls biofilm formation under phosphate limited conditions (Lamarche et al., 2008). CpxR (CDS56277.1), a member of the two-component regulatory system CpxA/CpxR has also been detected. In *E. coli*, the CpxA/CpxR system is required for settling biofilm communities and ensuring optimal cell-to-cell interactions (Otto & Silhavy, 2002; Beloin et al., 2004). Once activated, this strategic signaling pathway increases bacterial resistance to various environmental parameters, such as high pH conditions and antibiotics (Dorel, Lejeune & Rodrigue, 2006). Finally, the two-component regulatory system OmpR/EnvZ (CDS56155.1 and CDS56154.1) has also been detected by our proteomic approach. In *E. coli*, this system can mediate signal transduction in response to osmotic stress (Cai & Inouye, 2002) and can be involved in bacterial attachment to host epithelium via curli fibrils and in the regulation of genes associated with the invasiveness of pathogens and symbionts (Tabatabai & Forst, 1995).

### Quorum sensing

Defined as the ability of bacteria to monitor cell density before expressing a phenotype (e.g., biofilm formation or virulence) (Whitehead et al., 2001), quorum-sensing can play an essential role in symbiont or pathogen-host interactions. Quorum sensing allows invasive bacteria to adapt to the changing conditions found in their new niche and to resist various environmental stresses (e.g., nutritional and oxidative stress) associated with host-mediated responses. Our proteomic approach detected SwrI (CDS56499.1), an homologue of the autoinducer LuxI that has been already depicted as a key effector for host colonization in several symbiosis models (Visick et al., 2000; Pontes et al., 2008). This acyl-homoserine-lactone synthase catalyzes the synthesis of *N*-acyl-L-homoserine lactones (AHLs) that accumulates extracellularly as cell density increases. SwrR (the homologue of LuxR) has not been identified. In several *Serratia* species, the SwrI/SwrR quorum sensing system regulates diverse phenotypes such as swarming motility, the production of extracellular enzymes and antibiotics, and the formation of biofilms (Van Houdt, Givskov & Michiels, 2007). Whether *S. symbiotica* cells use cell–cell communication to monitor their population density via the synchronization of their behavior, or to socially interact during host colonization, remains to be investigated.

### Other proteins potentially involved in host invasion

We identified a series of other effectors potentially involved in adhesion, invasion and biofilm formation: Crp (CDS56109.1), which can repress biofilm formation (Jackson et al., 2002), Hns (CDS58425.1), which reduces bacterial adhesion in anoxic conditions by modulating the expression of flagellar genes (Landini & Zehnder, 2002), and the RNA polymerase sigma factor RpoS (CDS55654.1), which has a regulatory role in biofilm development (Sheldon et al., 2012). We identified the outer membrane protein A OmpA (CDS57861.1), known to participate in various pathogenic processes such as adhesion, invasion, biofilm formation and evasion of host defense (Smith et al., 2007; Martinez et al., 2014), and which is required for gut colonization by *S. glossinidius* in tsetse flies (Maltz et al., 2012). Finally, our proteomics analysis found the expression of the FKBP-type peptidyl-prolyl cis-trans isomerase FkpA (CDS56095.1), a protein that has been depicted as being similar to the macrophage infectivity potentiator (Mip) proteins of *Legionella pneumophila* and *Chlamydia trachomatis* (Lundemose, Kay & Pearce, 1993), and that contributes to intracellular survival of some members of the Enterobacteriacae (Horne et al., 1997).

### Iron acquisition

**Figure 3:**
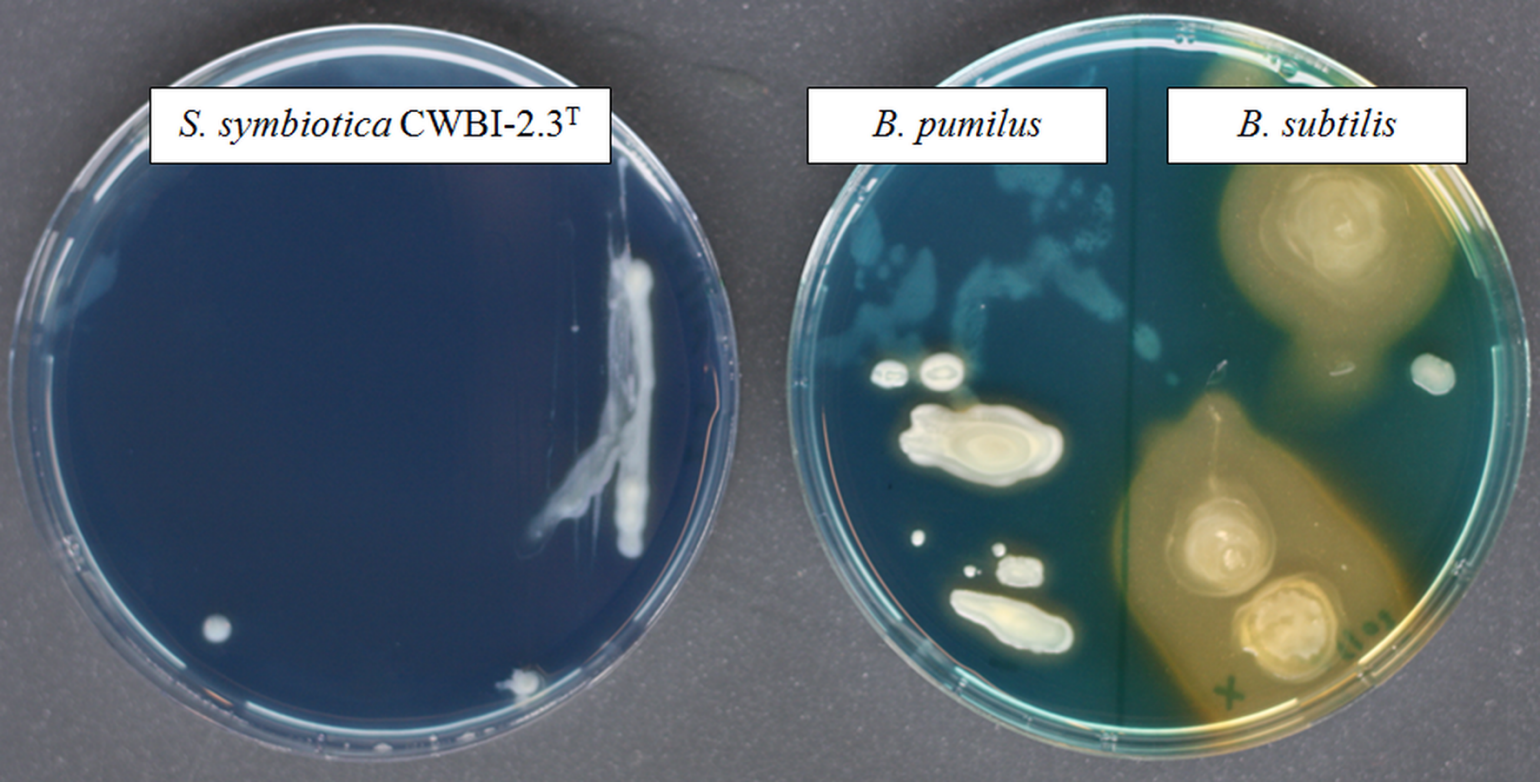
The screening of siderophore production *by S. symbiotica* CWBI-2.3T on CAS agar plates after 48 h of growth at 20°C as described in Materials and methods. *S. symbiotica* is negative for siderophore production. *B. pumilus* was used as negative control and *B. subtilis* was used as positive control.

Iron is a vital nutrient for growth of many bacterial species since it is used as a cofactor or as a prosthetic group for essential enzymes involved in many cellular functions (Schaible & Kaufmann, 2004). The availability of this element to bacteria within the host environment is generally limited. The successful uptake of iron is necessary for bacterial growth and virulence and a wide diversity of iron transport systems can be set up by invasive bacteria depending on iron state in the host environment (Braun & Hantke, 2011). With our proteomic approach, we successfully identified ExbB (CDS55934.1) and ExbD (CDS55935.1), two components of the cytoplasmic membrane-localized TonB-ExbB-ExbD complex which is required for full virulence and symbiosis in several invertebrate-bacteria interactions (Watson et al., 2010). No siderophore was detected in our proteome; however, we identified the transcriptional repressor Fur (CDS57198.1) which plays a key role in the regulation of siderophore biosynthesis and iron transport (Escolar, Pérez-Martín & De Lorenzo, 1999). A functional siderophore system enables pathogens and symbionts to scavenge iron under limiting conditions in symbiosis (Braun & Hantke, 2011; Han et al., 2013). By using a universal chemical assay, we found that *S. symbiotica* CWBI-2.3^T^ is negative for siderophore production (Fig. 3), indicating that the symbiont does not excrete siderophore in *in vitro* conditions. The ABC transporter Yfe, that is required for host colonization by invasive bacteria (Watson et al., 2010), was also detected with two identified proteins: YfeA (CDS58219.1) and YfeB (CDS58218.1).

The iron accumulated in the cytoplasm is often bound by specialized proteins such as ferritin and bacterioferritin. We successfully identified two ferritins: the ferritin iron storage protein Ftn (CDS58146.1), and Dps (CDS57357.1), which is a ferritin-like protein that can protect DNA from oxidative damage by sequestering intracellular Fe^2+^. Other iron-using proteins have been identified: the thiol peroxidase Bcp (CDS57424.1), the two succinate deshydrogenase SdhA (CDS57210.1) and SdhB (CDS57211.1), and the Fe(3+) dicitrate transport protein FecA (CDS55609.1). In the context of symbiosis, proteins involved in iron metabolism can have two functions: (i) enabling the bacterium to scavenge iron required for its own metabolism; (ii) countering host defenses that include the production of ROS for which iron is a precursor.

### Protection against ROS

The evolution of symbioses can be influenced by the oxidative homeostasis (i.e., the balance between reactive oxygen species (ROS) and antioxidant molecules) (Moné, Monnin & Kremer, 2014; Monnin et al., 2016). Indeed, ROS are toxic effectors that can participate in the host immune defense to regulate symbiont populations. To cross the host oxidative environment and protect themselves against ROS, invasive bacteria have developed a wide range of defenses. Our proteome analysis indicates that *S. symbiotica* is equipped with several proteins that are involved in the direct detoxification of ROS (Imlay, 2003). That includes the superoxide dismutase SodA (CDS56813.1), the catalase KatA (CDS57987.1), the thiol peroxidase Tpx (CDS58350.1) and the peroxiredoxin Bcp (CDS57424.1). We also identified five proteins (HemB, CDS56723.1; HemC, CDS56648.1; HemX, CDS56650.1; HemL, CDS55589.1; HemY, CDS56651.1) that belong to a cluster of seven proteins involved in the biosynthesis of protoporphyrin IX, the non-ferrous precursor of heme that is essential for the functionality of the enzymes involved in cellular protection against toxic oxygen radicals.

The proteome of *S. symbiotica* CWBI-2.3^T^ also includes several proteins involved in disulfide reduction such as the putative thioredoxin YbbN (CDS58953.1), the glutaredoxin YdhD (CDS58265.1), the glutathione reductase Gor (CDS56214.1), the electron transport complex subunit C RsxC (CDS58281.1), the thioredoxin reductase TrxB (CDS57678.1), and the thioredoxin TrxA (CDS56739.1). We also identified OxyR (CDS56327.1), the positive regulator of hydrogen peroxide inducible genes (Bauer, Elsen & Bird, 1999).

In addition to this battery of defense mechanisms against oxidative stress, *S. symbiotica* CWBI-2.3^T^ also harbors a set of chaperones for protein refolding and maturation such as ClpB (CDS59016.1), DnaK (CDS55519.1), GrpE (CDS58735.1), HtpG ( CDS58965.1) and NfuA (CDS56165.1) that are known to be involved in the repair of cell components following ROS mediated damage (Takemoto, Zhang & Yonei, 1998; Kitagawa et al., 2002; Hossain & Nakamoto, 2003; Angelini et al., 2008; Matuszewska et al., 2008).

### Secretion and transport mechanisms

Protein secretion plays a central role in modulating the interactions between bacteria with their environment, especially in pathogenic or symbiotic bacteria/host interactions. In our study, we identified several components of the general secretion pathway (Sec) and the two-arginine translocation pathway (Tat): the protein translocase subunit SecA (CDS55578.1), the chaperone SecB (CDS56816.1), SecD (CDS58907.1), SecF (CDS58906.1), SecG (CDS56777.1), SecY (CDS56065.1), and the sec-independent protein translocase, TatA (CDS56720.1). These two pathways are responsible for the transport of many proteins such as virulence factors and cell appendixes, across the outer membrane of Gram-negative bacteria (Natale, Brüser & Driessen, 2008). YajC (CDS58908.1) and YidC (CDS56458.1), that stabilize the insertion of SecA and its bound preprotein into the inner membrane, were also identified and form with SecD and SecF the Sec protein secretion pathway.

We identified the Type II secretion system (T2SS) protein G (CDS55483.1), suggesting that *S. symbiotica* CWBI-2.3^T^ could be provided by a secretion system only found in proteobacteria and that can be found in symbiotic bacteria as well as pathogens (Costechareyre et al., 2013). The T2SS secretes various virulence determinants and has been shown to be important for virulence in many pathogens (Cianciotto, 2005). A recent study has demonstrated that the T2SS is essential for gut colonization by the leech digestive tract symbiont *Aeromonas veronii* (Maltz & Graf, 2011). The presence of T2SS is intriguing, since most of the secretion systems described in insects symbionts belongs to Type III and Type IV secretion systems (Dale & Moran, 2006; Degnan et al., 2009; Oliver et al., 2010). This T2SS has also been described as an important virulence factor of a number of gram-negative bacterial plant pathogens (Jha, Rajeshwari & Sonti, 2005). Its potential presence in *S. symbiotica* CWBI-2.3^T^ suggests that the bacterium could be or could have been a plant pathogen. This hypothesis is supported by the detection of a non-ribosomal peptide synthetase (CDS58510.1) that could be a fragment of the syringopeptin synthetase C, an hydrolase that has been described as a virulence factor in some plant pathogens (Scholz-Schroeder et al., 2001). Syringopeptin is a known necrosis-inducing phytotoxin and therefore raises the question of the origin of symbionts; it is possible that *S. symbiotica* was originally a plant pathogen that has been acquired by aphids feeding on infected host plants, and then gradually domesticated by these insects. Experiments are being conducted to investigate this hypothesis

Remarkably, two structural components of a multi-drug resistance efflux pump belonging to the resistance nodulation division family were also identified: the perplasmic protein AcrA (CDS58973.1) and the outer membrane channel protein TolC (CDS55974.1). *S. symbiotica* CWBI-2.3^T^ exhibits a resistance to vancomycin (Sabri et al., 2011). Whether these antibiotics are indeed exported by an AcrAB-TolC efflux pump system remains to be tested.

### Hydrolytic enzymes

Invasive bacteria rely on proteolysis for a variety of purposes during the infection process. Several enzymes with potential proteolytic and chitinolytic activity were identified in our proteome analysis. ClpB (CDS59016.1), ClpX (CDS58879.1), ClpP (CDS58880.1), and the Lon protease (CDS58878.1) are the main proteolytic players in the cytosol and can contribute to bacterial virulence (Frees, Brøndsted & Ingmer, 2013). We also identify serine endoproteases DegP (CDS55594.1) and DegQ (CDS55928.1). *S. symbiotica* CWBI-2.3^T^ also contains several metalloproteases such as YaeL (CDS58788.1) and FtsH (CDS56781.1). Finally, we identified one chitodrexinase (CDS55463.1) involved in the pathway of the degradation of chitin, a characteristic component of the cell walls of fungi and the exoskeletons of arthropods. The identification of chitinases, which may possess antifungal activity, may contribute to the protection of the aphid host against fungal pathogens as observed in several cases of symbiosis (Łukasik et al., 2013). This question should be addressed in further experiments.

## Concluding Remarks

The comprehensive proteome analysis of the free-living strain *S. symbiotica* CWBI-2.3^T^ resulted in the identification of 720 proteins corresponding to 19.7% of all theoretically expressed proteins of the symbiont. Most of the identified proteins belong to housekeeping and primary metabolism. Special attention was drawn to putative symbiotic factors and the most striking were: (i) members of the two components signaling systems that are no longer expressed in obligate symbionts and several facultative ones, (ii) OmpA, which is a probable key factor for symbiosis establishment, (iii) FkpA that could contribute to the intracellular survival of *S. symbiotica*, (iv) iron transport mechanisms to access host iron resources, and (v) several components of secretions systems which might be involved in the secretion of still unknown effectors. In addition, *S. symbiotica* CWBI-2.3^T^ is equipped with a wide range of protections against the hostile oxidative environment of the host. These results provide strong evidence that *S. symbiotica* CWBI-2.3^T^ is well-armed to invade, persist, and multiply in the insect tissues. Interestingly, we did not find any pathogenic trait that would directly harm the host such as toxins. Moreover, swimming motility and chemotaxis, which can be determinant for host colonization, are missing in the free-living *S. symbiotica* CWBI-2.3^T^.

Phylogenetic analyses combined with fluorescence *in situ* hybridization approaches applied on *S. symbiotica* strains harbored by various aphid species reveal that certain strains naturally reside in the aphid gut (Renoz et al. in prep). Interestingly, *S. symbiotica* strain CWBI-2.3^T^ belongs to the same clade as these extracellular gut symbionts, suggesting that it could be picked up directly from environmental sources, and thus initiate novel symbiotic associations with the insect digestive tract as the first potential entry route. Evidence from our study suggests that *S. symbiotica* CWBI-2.3^T^ could be, initially, a plant pathogen. The pathogenicity of *S. symbiotica* to plants, as well as the role of plants as mediator of horizontal transfers of the symbiont, are currently under investigation.

Our proteomic analysis was carried out from bacteria maintained in *in vitro* conditions that do not reflect the conditions actually encountered in nature. Consequently, it is important to bear in mind that the adaptation of this *S. symbiotica* strain to *in vitro* conditions may have altered certain of its physiological characteristics, and therefore proteins such as pathogenic and symbiotic factors whose expression may differ under more relevant ecological conditions (Fux et al., 2005).

There is no doubt that free-living strains of *S. symbiotica* will constitute an experimental tool for understanding the manifold strategies set up by symbiotic bacteria during the nascent phase of a symbiosis in order to ensure their long-term establishment in a novel insect host. This comprehensive proteome map of a facultative insect symbiont provides a series of potential symbiotic factors for further studies to understand the molecular cross-talk between aphids and *S. symbiotica* of ecological and evolutionary importance.

##  Supplemental Information

10.7717/peerj.3291/supp-1 Table S1 Supporting Information Table S1

## Additional Information and Declarations

### Data Availability

The following information was supplied regarding data availability: The raw data has been supplied as Table S1.
